# Gastroenteritis outbreaks on cruise ships: contributing factors and thresholds for early outbreak detection

**DOI:** 10.2807/1560-7917.ES.2017.22.45.16-00576

**Published:** 2017-11-09

**Authors:** Varvara A Mouchtouri, Eleni Verykouki, Dumitru Zamfir, Christos Hadjipetris, Hannah C Lewis, Christos Hadjichristodoulou, the EU SHIPSAN ACT partnership

**Affiliations:** 1 Department of Hygiene and Epidemiology, Faculty of Medicine, University of Thessaly, Larissa, Greece; 2 Celestyal Cruises, Piraeus, Greece; 3 Robert Koch Institute, Berlin, Germany; 4 The members of the group are listed at the end of the article

**Keywords:** maritime, ship, travel, health, gastroenteritis, threshold, norovirus, cruise

## Abstract

When an increased number of acute gastroenteritis (AG) cases is detected among tourists staying at the same accommodation, outbreak management plans must be activated in a timely manner to prevent large outbreaks. Syndromic surveillance data collected between 1 January 2010 and 31 December 2013 by five seagoing cruise ships were analysed to identify attack rate thresholds for early outbreak detection. The overall incidence rate of AG was 2.81 cases per 10,000 traveller-days (95% confidence interval (CI): 0.00–17.60), while the attack rate was 19.37 cases per 10,000 travellers (95% CI: 0.00–127.69). The probability of an outbreak occurring was 11% if 4 per 1,000 passengers reported symptoms within the first 2 days of the voyage, and this increased to 23 % if 5 per 1,000 passengers reported such within the first 3 days. The risk ratio (RR) for outbreak occurrence was 2.35, 5.66 and 8.63 for 1, 2 and 3 days’ delay of symptoms reporting respectively, suggesting a dose–response relationship. Shipping companies’ policies and health authorities’ efforts may consider these thresholds for initiating outbreak response measures based on the number of cases according to day of cruise. Efforts should focus on ensuring travellers report symptoms immediately and comply with isolation measures.

## Introduction

Acute gastroenteritis (AG) is the most frequent disease among travellers and outbreaks are detected among tourists staying in accommodation every year [1], including on cruise ships [2]. The majority (97%) of AG outbreaks that were reported and diagnosed on cruise ships in the United States (US) during 2008–2014 were caused by norovirus [2]. Norovirus gastroenteritis outbreaks often involve person-to-person and environmental transmission [3-9] and have been recognised to have a considerable public health impact and economic burden on the tourism industry [10,11]. In addition to the public health impact and disruption of holidays, consequences include loss of personal travel funds and high ship operation costs [10]. Preventative routine measures include pre-embarkation screening, syndromic surveillance, isolation of symptomatic travellers (i.e. crew members and passengers), environmental and personal hygiene measures, crew education, and instructions to travellers and others about hand washing and symptoms reporting [12,13].

Cruise ships implement syndromic surveillance of AG cases using standard definitions, as described in guidelines from the World Health Organization (WHO) [14], and as required by national and international inspection programmes for cruise ships [12,13]. For example, the European Manual for Hygiene Standards and Communicable Disease Surveillance on Passenger Ships includes a chapter for the prevention and control of AG, and recommends standards for syndromic surveillance and outbreak management plans on passenger ships sailing in European Union (EU) countries’ waters [12]. Moreover, this manual suggests that pre-defined thresholds for outbreak alert reports and that control measures should be agreed and included in the outbreak management plan. When increased numbers of AG cases are diagnosed in the ships infirmary, outbreak management plans are activated, intensifying the routine prevention measures and implementing additional ones, such as active surveillance, enhanced environmental disinfection, discontinuing the self-service buffet, and social distancing [12,13]. Activating the outbreak management plans in a timely manner is important and can help to prevent the further person-to-person transmission that results in large outbreaks [12,13]. However, the optimal time for initiating intensive measures and activating the outbreak management plan is under discussion and requires further research.

The International Health Regulations (IHR) 2005 requires ship captains to notify the port public health authority of any public health risk on board a vessel before arrival at the port [15]. The port health authorities are responsible for checking that valid health and hygiene plans, including outbreak management plans, are in place, when notified of a risk on board. Shipping companies have different thresholds for initiating implementation of certain control measures in their plans. For example, outbreak plan activation may occur when there are six AG cases within 6 hours, 1% of passengers affected with AG on ships with less than 1,000 passengers or 0.5% affected on ships with more than 1,000 passengers [12]. Some port health authorities require submission of outbreak reports by a vessel’s captain when certain thresholds are reached (e.g. first report when 2% of AG cases among passengers or 2% among crew and a second report when 3% of AG cases among passengers or 3% among crew), but this threshold might be too high if used for determining the activation of outbreak management plans. A study by Wikswo et al. reported that infirmary surveillance detected only 60% of AG cases in an outbreak and suggest that adjustments to outbreak reporting thresholds may be needed to account for incomplete voluntary AG reporting and to more rapidly implement control measures [16]. The US Centers for Disease Control and Prevention (CDC) Vessel Sanitation Programme (VSP) suggests that a 0.45% daily attack rate of AG on ships is indicative of a pending outbreak [13].

The objectives of this study were: (i) to analyse syndromic surveillance data of AG on cruise ships in order to determine AG cases’ characteristics, incidence and attack rates for cruises with and without outbreaks, (ii) to identify thresholds marking levels of reported AG cases at which an outbreak is likely to occur at and (iii) to identify risk factors associated with outbreaks and determine possible response measures.

## Methods

Data on cases recorded in the AG surveillance logs of five seagoing cruise ships belonging to a single cruise line that conducted 760 cruises between 1 January 2010 and 31 December 2013 were collected and analysed. The countries that the cruise ships visited were Cuba, Cyprus, Dominican Republic, Egypt, France, Greece, Italy, Portugal, Spain and the United Kingdom. The five ships implemented the same policies for surveillance as well as health and hygiene, including outbreak prevention and management, in the 4-year period. This meant they followed the same rules, data collection, case definitions and health and hygiene management plans.

The cruise ships’ policies comply with the European Union (EU) legislation described in the European Manual for Hygiene Standards and Communicable Disease Surveillance on Passenger Ships, and the US VSP, both of which incorporate the global standards of WHO guidelines for ship sanitation [12-14,17]. Pre-embarkation screening occurred through passenger completion of a one-page questionnaire on certain symptoms prior to boarding. In terms of hand hygiene, informative leaflets with hand hygiene advice were distributed (pillow letter), there were signs posted in various areas on board, including at the buffet area entrances, in dining rooms, in the toilet facilities and around hand washing sinks, that reminded passengers and crew to wash their hands often. Crew members received initial and regular refresher training on health and hygiene issues, and a crew member was responsible for ensuring that all persons entering the buffet areas and dining rooms used hand antiseptic. Those measures intensified during outbreaks. All AG cases were given verbal and written advice on hand hygiene.

### Acute gastroenteritis case and outbreak definition

The ship medical staff routinely recorded any traveller, which refers to both passengers and crew members, fulfilling the following criteria into the AG log: acute diarrhoea (three or more episodes of loose stools in a 24-hour period) or vomiting and at least one of the following: one or more episodes of loose stools in a 24-hour period, abdominal cramps, headache, muscle aches or fever as diagnosed by the ship medical doctor [12,13]. Medical consultations to AG cases, as well as laboratory examinations were provided free of charge.

Stool samples were collected from travellers fulfilling the definition of an AG case and were analysed in a laboratory ashore. The RIDASCREEN Norovirus 3rd Generation test was used, which is a qualitative enzyme immunoassay intended for the detection of selected genogroup I and genogroup II norovirus strains in human faeces. Specimens were taken and transported in accordance with the manufacturer’s instructions (R-Biopharm AG, Darmstadt, Germany). In addition to norovirus tests, stool specimens were examined by culture for *Salmonella* spp., *Shigella* spp., *Staphylococcus aureus*, and enteropathogenic Escherichia *coli*.

The data in the AG log for each cruise contained information about cruise dates, name of all ports of call, number of passengers and crew numbers, number of travellers with AG symptoms and their age and sex, whether they were passengers or crew members, their cabin number, meal seat in the dining room and the date and time of symptom onset. Symptom data recorded in the AG log included absence or presence of diarrhoea and number of diarrhoea episodes, presence or absence of bloody diarrhoea, presence or absence of vomiting and number of vomiting episodes, and presence or absence of fever and temperature measurement results that were recorded in the AG log. Information on whether any stool samples were taken and if those samples were examined for norovirus and various bacterial pathogens together with their result were also recorded.

‘Outbreak cruises’ were considered those where ≥ 2% of passengers or ≥ 2% of crew members reported AG symptoms. ‘Non-outbreak cruises’ were considered those where < 2% of passengers and < 2% of crew reported AG symptoms. AG log data from contiguous cruises immediately after an outbreak were excluded from the analysis because of the potential for the source of the outbreak to be associated with that of previous cruise. Overall attack rates and incidence rates were calculated per 10,000 travellers and per 10,000 traveller-days respectively. Moreover, attack rates and incidence rates were calculated for cruises with less than 7 days and for more than 7 days, as well as for non-outbreak cruises and for outbreak cruises. Attack rates per 10,000 passengers were calculated for cruises per the country of home port (i.e. port where most passengers disembark and new passengers embark).

### Statistical analysis

Data analysis included descriptive statistics and univariate analyses. Risk ratio (RR) was used to describe the association between outbreak occurrence and the duration of symptoms at time of reporting of AG cases. The Receiver Operating Characteristic (ROC) analysis was used for assessing the diagnostic accuracy of the attack rate of passenger AG cases occurring during the first 2–3 days of a cruise and for determining the optimal cut-off point of the attack rate that can be applied to decision-making [18]. Generalised estimating equations (GEE) models for logistic regression that are used for the analysis of longitudinal data were applied to estimate the daily probability of an outbreak. GEE models allow dependence within clusters. All p values less than 0.05 were considered statistically significant. Analysis was conducted using R version 3.0.2 Statistical Software (R Foundation for Statistical Computing, Vienna, Austria).

Threshold levels were calculated based on passenger-days and reported in per 1,000 passengers because all outbreaks in the study occurred among passengers and not crew. Threshold levels were displayed graphically to show the minimum number of travellers with AG symptoms required for an outbreak to occur. The levels were obtained by the multiplication of the incidence rate (per 10,000 passenger-days), the number of days on board, and the number of the passengers that a cruise may have on a journey divided by 10,000 passenger-days.

Passenger and crew member numbers were considered stable throughout each cruise (i.e. it was assumed that no one left or got on during a single cruise). Study exiting or entering during a single cruise were therefore not considered in the calculation of traveller-days (i.e. passengers and crew) and passenger-days.

## Results

### Descriptive analysis

A total of 760 cruises, varying in size from 1,250 to 4,000 travellers were included in the analysis. Cruise length ranged from 3 to 20 days, with the majority (56.1%) lasting 7 days. The total number of traveller days was 7,791,657 and the total number of passengers was 869,704. Table 1 presents the characteristics of cases including age and sex. Age information was available for the 1,932 cases. A total of 55 cases among passengers (2.9%) were children aged < 15 years. Children aged < 15 years comprised 2.6% (n = 13/495) of the total number of cases in cruises with an outbreak (95%CI: 1.44–4.45) and 2.9% (n = 42/1437) in cruises without one (95% CI: 2.11–3.93).

**Table 1:** Characteristics of acute gastroenteritis cases on cruise ships and frequency of symptoms by categories of travellers, Cuba, Cyprus, Dominican Republic, Egypt, France, Greece, Italy, Portugal, Spain and the United Kingdom, 2010–2013 (n = 1,936)

Characteristic/ symptom of AG cases	All travellers		Passengers		Crew members	
	n or (mean)	% or (SD)	n or (mean)	% or (SD)	n or (mean)	% or (SD)
Age, years	(55.2)	(18.3)	(57.9)	(17.1)	(32.1)	(9.2)
Females	1,069	55.3	995	57.6	74	35.9
Diarrhoea^a^	1,815	93.7	1,616	93.6	199	96.6
Including bloody diarrhoea^a^	30	1.6	26	1.5	4	1.9
Diarrhoea episodes per 24 hours	(5.3)	(3.6)	(5.4)	(3.7)	(4.4)	(2.7)
Vomiting^a^	1,207	62.4	1,097	63.5	110	53.6
Vomiting episodes^a^	(3.9)	(3.2)	(4.1)	(3.3)	(2.9)	(2.2)
Fever^b^	190	9.8	175	10.1	15	7.2
Fever (°C)^c^	(38.4)	(0.42)	(38.4)	(0.41)	(38.3)	(0.52)

SD: standard deviation.

^a^ Reported by patient at the infirmary.

^b^ Presence as diagnosed by doctor.

^c^ Temperature as measured at the infirmary.

During the study period, nine outbreaks of AG occurred; all outbreaks occurred among passengers and in cruises with a duration of 7 days or more. A total of 1,936 AG cases were identified during the study period, with the majority (n = 1,727, 89.2%) of those occurring among passengers and the remaining occurring among the crew members.

The majority of cases presented with diarrhoea (93.7%), while 62.4% presented with vomiting and 9.8% presented with fever (Table 1). Of the 1,936 cases, 1,439 (74.3%) were examined for norovirus and bacterial pathogens. Of the 1,439 cases, 660 (45.8%) tested positive for norovirus while all tested negative for bacteria test results. There were at least two norovirus-positive cases for eight outbreaks, while there were no laboratory results available for one outbreak.

### Incidence rates and attack rates

The overall incidence rate was 2.81 cases per 10,000 traveller-days (95% CI: 0.00–17.60) while the attack rate was 19.37 cases per 10,000 travellers (95% CI: 0.00–127.69). For cruises with a duration of 7 or more days, the overall incidence rate was 3.99 cases per 10,000 traveller-days (95% CI: 0.00–22.53) and the attack rate was 30.09 cases per 10,000 travellers (95% CI: 0.00–169.43). The attack rate was 14.05 (95% CI: 0.00–55.08) per 10,000 travellers for non-outbreak cruises and 238.80 (95% CI: 0.00–738.70) per 10,000 travellers for outbreak cruises (Table 2). The incidence rate was 2.13 (95% CI: 0.00–8.12) per 10,000 traveller-days for non-outbreak cruises and 31.04 (95% CI: 0.00–100.94) per 10,000 traveller-days for outbreak cruises. Considering non-outbreak cruises with a duration of 7 or more days, the overall incidence rate was 2.90 (95% CI: 0.00–9.88) cases per 10,000 traveller-days while the attack rate was 21.51 (95% CI: 0.00–71.90) cases per 10,000 travellers.

**Table 2:** Incidence rates of acute gastroenteritis per 10,000 traveller-days and attack rates for cruises with and without an outbreak, Cuba, Cyprus, Dominican Republic, Egypt, France, Greece, Italy, Portugal, Spain and the United Kingdom, 2010–2013 (n = 760 cruises)

Year	Cruises without an outbreak				Cruises with an outbreak			
	IR (cases/10,000 traveller-days)	95% CI	AR (cases/10,000 travellers)	95% CI	IR (cases/10,000 traveller-days)	95% CI	AR (cases/10,000 travellers)	95% CI
Overall	2.13	0.00–8.12	14.05	0.00–55.08	31.04	0.00–100.94	238.80	0.00–738.70
2010	2.85	0.00–9.74	21.28	0.00–72.05	17.95	0.00–54.22	169.08	0.00–571.24
2011	2.19	0.00–8.00	14.24	0.00–54.31	46.46	0.00–140.19	325.19	0.00–981.33
2012	1.67	0.00–6.67	10.22	0.00–39.98	9.48	0.00–28.07	138.06	0.00–428.50
2013	1.96	0.00–8.17	12.08	0.00–53.27	26.11	0.00–53.56	191.79	0.00–388.36

CI: confidence interval; IR: incidence rate.

The attack rate for cruises with home ports within Europe was 2.77 cases per 10,000 passengers, while the attack rate for cruises with home ports outside Europe was 7.04 cases per 10,000 passengers. The highest attack rates were recorded in cruises with home ports in Cuba and Egypt (Table 3). The rate ratio was 2.54 (95% CI: 2.24–2.87), indicating that cruises with home ports outside Europe had 2.5 times higher attack rates than cruises with home ports within Europe.

**Table 3:** Attack rates of acute gastroenteritis per 10,000 passengers for cruises by home port, 2010–2013 (n = 760)

Country of home port^a^	Number of cruises (n)	Number of AG cases (n)	Total passengers (n)	Cases per 10,000 passengers
**Non-European Union countries**				
Cuba	2	6	330	181.82
Dominican Republic	33	149	47,116	31.62
Egypt	9	167	10,782	154.89
**European Union countries**				
Cyprus	69	212	62,986	33.66
France	2	0	1,709	0.00
Greece	412	579	431,479	13.42
Italy	85	197	109,761	17.94
Portugal	1	10	1,286	77.76
Spain	117	518	167,246	30.97
United Kingdom	30	98	37,009	26.48

AG: acute gastroenteritis

^a^ The port where changeover of the majority of passengers takes place.

### Duration of symptoms at time of reporting

From the total 1,936 AG cases, 614 (31.7%) had symptoms for 1 day, 107 (5.5%) for 2 days and 78 (4.0%) for 3 days or more at the time of reporting. The remaining cases reported their symptoms the day they occurred. Duration of symptoms at time of reporting (i.e. delay in reporting) was not associated with sex (p > 0.05) or age (p > 0.05). The risk of an outbreak occurring was 2.3 times higher in cruises where one or more passengers delayed reporting their symptoms for 1 or more days (RR: 2.35, 95% CI: 2.16–2.55, p < 0.001) (Table 4). The RR for outbreak occurrence was 5.66 for a symptoms reporting delay of 2 or more days and 8.63 for 3 or more days. This suggests a dose–response relationship.

**Table 4:** Risk ratio of the occurrence of an outbreak according to length of delay in acute gastroenteritis symptoms reporting by passengers, Cuba, Cyprus, Dominican Republic, Egypt, France, Greece, Italy, Portugal, Spain and the United Kingdom, 2010–2013

Duration of symptoms at the time of reporting in days according to the day of cruise	All cruises (n = 760)			Cruises ≥ 7 days long (n = 427)		
	RR	95% CI	p value	RR	95% CI	p value
One or more passengers symptomatic for 1 day before reporting symptoms at any time of the cruise	2.35	2.16–2.55	< 0.001	1.90	1.75–2.06	0.005
One or more passengers symptomatic for 2 days before reporting symptoms at any time of the cruise	5.66	4.26–7.52	< 0.001	4.50	3.37–6.02	< 0.001
One or more passengers symptomatic for ≥ 3 days before reporting symptoms at any time of the cruise	8.63	5.11–14.58	< 0.001	7.03	4.12–12.03	< 0.001
One or more symptomatic passengers without reporting symptoms during the first 2 days of the cruise	2.45	0.96–6.29	0.088	2.23	0.87–5.76	0.128
One or more symptomatic passengers without reporting symptoms during the first 3 days of the cruise	3.84	2.94–5.00	< 0.001	3.55	2.70–4.69	< 0.001

CI: confidence interval; RR: risk ratio.

### Outbreak probability and threshold determination

For the ROC analysis, the optimal cut-off point for each curve was found to be equal to one case per 1,000 passengers and two cases per 1,000 passengers for the first 2 and 3 days, respectively (Table 5). When all cruises were taken into account, the probably of an outbreak occurring was 11.1% if four cases per 1,000 passengers reported AG symptoms in the first 2 days of a cruise and 23.1% if there were five AG cases per 1,000 passengers the first 3 days of the cruise. Only considering cruises with a length of 7 or more days, these probabilities increase to 12.5% and 33.4%, respectively.

**Table 5:** Probability of outbreak occurrence according to the number of acute gastroenteritis cases per 1,000 passengers during the first two and three days of a cruise, Cuba, Cyprus, Dominican Republic, Egypt, France, Greece, Italy, Portugal, Spain and the United Kingdom, 2010–2013

Number of acute gastroenteritis cases per 1,000 passengers	All cruises (n = 760)			Cruises ≥ 7 days long (n = 427)		
	Probability of an outbreak (PPV, %)	ROC area	95% CI	Probability of an outbreak (PPV, %)	ROC area	95% CI
**First two days of the cruise**						
1	4.63	0.74	0.55–0.93	6.49	0.75	0.54–0.92
2	6.82			8.82		
3	6.68			7.69		
4	11.07			12.51		
**First three days of the cruise**						
1	3.50	0.87	0.72–1.00	4.83	0.87	0.71–1.00
2	7.61			9.73		
3	14.64			17.14		
4	22.76			25.03		
5	23.10			33.37		

PPV: positive predictive values; ROC: Receiver Operating Characteristic

The graph presents a way of showing the number of AG cases, according to cruise size and day of the cruise, at or above which an AG outbreak is probable (Figure). For example, if there is a cruise ship of 2,000 people, the cruise is on day 20, and there are 24 people reporting symptoms, an outbreak is probable.

**Figure:**
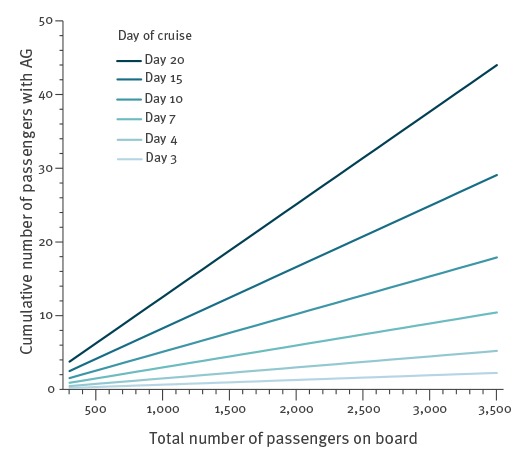
Number of AG cases on board a cruise ship by cruise size and day at or above which an AG outbreak is probable, Cuba, Cyprus, Dominican Republic, Egypt, France, Greece, Italy, Portugal, Spain and the United Kingdom, 2010–2013

AG: acute gastroenteritis.

## Discussion

Our retrospective study was the first attempt of the EU SHIPSAN ACT joint action [19,20] to cooperate with a cruise line and produce data-driven thresholds for outbreak prevention based on infirmary surveillance data. They can be used by ship operators to inform them when enhanced control measures should be initiated in their outbreak management plans. These thresholds can also be used by public health authorities for risk assessment and decision-making in terms of whether the number of reported cases is within the expected levels or not.

The collaboration of the cruise industry with the US CDC VSP resulted in lower numbers and severity of cruise ship AG outbreaks between 2008–2014 compared with 2001–2004 [2,4]. The US CDC VSP receives reports from cruise ships with information about diarrheal disease cases 24–36 hours before arriving in the US from a foreign port and additional reports when the cumulative AG attack rate among either passengers or the crew is ≥ 2% and ≥ 3% [13]. Our study suggested lower triggers as indicators of a pending outbreak during the first three days of the cruise than that of the US CDC VSP manual. We calculated a 33.37% probability of having an outbreak if there are five AG cases per 1,000 passengers within the first three days of a cruise on cruises of 7 days or more while the US VSP manual suggests a 0.45% daily attack rate (or 4.5 cases per 1,000 passengers daily) is indicative of a pending outbreak [13]. In the future, it would be interesting to study surveillance data reported from different cruise lines in EU countries and compare those with other regions.

Incidence rates of AG in our study were higher in cruises with home ports outside Europe, i.e. Egypt and Cuba. It was not possible to examine if other factors such as season, passenger nationality or others affected this association. Moreover, this study did not show any indication that children could be the drivers of AG outbreaks on board ships.

In our study the overall incidence rate was 2.81 (95% CI: 0.00–17.60) cases per 10,000 traveller-days, while the attack rate was 19.37 cases per 10,000 travellers. These results are similar to the recently published report from the US where the overall AG attack rate reported by cruise ships in 2008–2014 was 1.8 cases per 1,000 passengers and the incidence rate of AG per 10,000 travel days ranged from 2.09 in 2013 to 2.72 in 2008 [2].

The outbreak frequency found in our study (9 outbreaks in 760 cruises; 1.18%) was higher than that from US surveillance data (133 outbreaks in 29,107 cruises; 0.45%), but these results cannot be directly compared since the definition of an outbreak differed (≥ 2% attack rate among passengers or among crew in our study, ≥ 3% attack rate among passengers or among crew in the CDC study [2]).

According to the laboratory results, and the clinical criteria, it seems that the nine AG outbreaks could have been caused by norovirus [21,22]. However, it should be noted that more than half of the cases examined for norovirus tested negative by immunochromatographic test for norovirus genogroups I and II. These results were not confirmed by a second molecular method, which might have increased the proportion of positive laboratory tests [22]. Notwithstanding this, the policy of the company to perform microbiological analysis of clinical specimens of patients for norovirus and bacteria aids early recognition of the aetiology of outbreaks and can be of value for informing decision making on adequate control measures.

Our study demonstrated that 31.7% of AG cases had symptoms for 1 day, 5.5% for 2 days and 4.0% for 3 days or more at the time of symptom reporting. In their study, Wikswo et al demonstrated that 40% of responders did not report symptoms to the infirmary during an outbreak with 236 case passengers [16]. Considering such high proportions of unreported cases during outbreaks, it could be assumed that the probabilities for an outbreak calculated by this study could be even higher. Our study also showed that the risk of an outbreak increased by 3.7-fold when there was a 3-day delay of symptoms reporting compared to 1-day delay. Future studies could examine the unreported cases on outbreak and non-outbreak cruises, and adjust the probabilities using assumptions for the numbers of unreported cases accordingly [16].

A retrospective survey conducted during a cruise ship norovirus outbreak showed that most cases delayed or did not report their illness to the ship’ s infirmary because they did not believe it was serious [23]. Denying of symptoms has even been reported, even if video surveillance data recorded public faecal accident and bringing of the towel stained with diarrhoea back to the pool towels bin [24]. The reasons for not reporting symptoms can be manifold and shipping companies’ policies should focus on communication strategies informing passengers about AG transmission and the importance of immediate symptoms’ reporting as well as the impact of AG outbreaks. Crew members can play an important role in prevention strategies, by complying with travel health and hygiene policies, and by actively monitoring and participating in their implementation. Furthermore, travel agents, travel health authorities can also help support creating awareness for AG-related issues and prevention.

One limitation of this study is that it analysed data from a single cruise line that implemented the same policy in all ships so it was not possible to compare epidemiological data from different companies. To address this, future studies should be conducted with data from more ships and from different companies and thresholds should be based on the number of cases and events. Other future studies should investigate the association of outbreaks with itineraries and season, with the purpose of identifying any risk or protective factors.

Another limitation was that we were not able to examine if other factors, such as the nationality of cases, have an effect on AG and specifically, norovirus infection. Future studies may further examine this factor. Cruise ships are a gathering place for many nationalities and ethnicities and it could be worthwhile surveying associations between these variables and risk for infection or outbreak occurrence. Also, it should be noted that in our study, the true number of symptomatic cases is unknown since only travellers who visited the ships infirmary were included in the AG log. Moreover, the role of asymptomatic travellers in the outbreak occurrence is not known.

In conclusion, this study confirmed the importance of aggregating and reviewing daily syndromic surveillance data of AG on cruise ships, particularly regarding the first days of the voyage, and demonstrated the impact of reporting delay on the occurrence of AG outbreaks on cruise ships. Finally, it provided specific numerical thresholds that should allow shipping companies and public health authorities to determine when an outbreak is occurring based on epidemiological evidence, and adjust/update their outbreak management planning accordingly.

## Notes

**EU SHIPSAN ACT joint action partnership:** Despena Andrioti, Anastasia Barbouni, Nikolaos Bitsolas, Janneke Broekhuijsen, Miguel Davila-Cornejo, Martin Dirksen-Fischer, Rita Maria Ferrelli, Andreas Gilsdorf, Irene Goverse, Brigita Kairiene, Antonis Kantonis, Galina Kokosharova, Elina Kostara, Jenny Kourea-Kremastinou, Leonidas Kourentis, Angel Kunchev, Audrone Lavruvianec, Peter Otorepec, Rimantas Jonas Pilipavicius, Martina Pilkova, George Rachiotis, Persa Tserkezou, Carmen Varela Martinez, Natalja Võzelevskaja.
